# A Pediatric Case of Benzodiazepine Poisoning Diagnosed Following the Appearance of a Brilliant Blue Tongue

**DOI:** 10.1155/crpe/8864772

**Published:** 2025-09-15

**Authors:** Yuri Hayashi, Takayuki Miyamoto, Manami Suzuki, Hiroki Sato, Atsumi Takechi, Shuji Fujino, Akiyoshi Takahashi, Tsutomu Watanabe

**Affiliations:** Department of Pediatrics, Tokushima Red Cross Hospital, 103 Irinokuchi Komatsushima-cho, Komatsushima 773-0001, Tokushima, Japan

**Keywords:** abuse, benzodiazepine, blue, drug, flunitrazepam

## Abstract

Benzodiazepines are one of the commonly used prescription anxiolytic drugs; however, they are increasingly used for drug abuse, drug crime, and sometimes for medical child abuse. To prevent misuse of high-potency benzodiazepines, some of them are currently manufactured as a tablet with a speckled blue core that dyes liquid blue when dissolved in drinks. Diagnosing drug poisoning, especially in cases of medical child abuse, can be challenging when signs of ingesting drugs, including empty medical packages, are missing. Herein, we report an infant's case of benzodiazepine poisoning, who was diagnosed with disturbed consciousness and a blue-colored tongue. An 11-month-old boy was referred to our hospital as his tongue was colored blue. According to his family, no blue-colored items were found around him when they noticed his tongue was blue. Physical examination revealed his consciousness was slightly disturbed. Benzodiazepine poisoning was suspected from his level of consciousness and blue-colored tongue, and it was detected using a urine drug test kit (SIGNIFY ER). Medical child abuse was suspected, as accidental ingestion was not likely to happen in the circumstances heard from his family members. Everyone around him denied having benzodiazepine, and how he ingested the medicine was not revealed despite intensive investigation by the police. Benzodiazepine poisoning should be considered in patients presenting with a blue tongue with disturbed consciousness. Adding dyes to medicines commonly used for poisoning may be helpful in recognizing and preventing child abuse.

## 1. Introduction

Benzodiazepines are clinically prescribed as depressants for various indications, including seizures, anxiety disorders, and insomnia. However, potent benzodiazepines, particularly flunitrazepam, which has a sedative effect 10 times as potent as that of diazepam, are preferred by drug abusers and are notorious for their connection to drug-facilitated sexual assaults. To prevent criminal use, flunitrazepam is currently manufactured as a tablet with a speckled blue core that dyes liquid blue when dissolved in drinks. Herein, we report a pediatric case of benzodiazepine poisoning, where the diagnosis was facilitated by a brilliant blue–colored tongue.

## 2. Case Presentation

**Figure 1:**
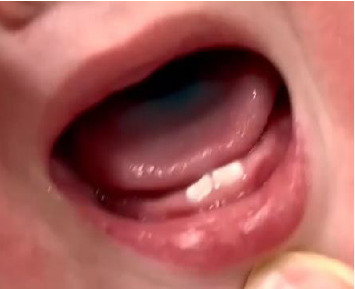
An image of the patient's brilliant blue–colored tongue.

An 11-month-old boy was transported to our hospital. He had been left alone in a playpen for several minutes, after which his grandmother noticed that his tongue had turned blue (Figure 1). No blue-colored items were located inside the playpen, and his mother and grandmother were unaware of what he had swallowed. They denied having any medication, and no packages of drugs were found in his vicinity. One hour had passed between the time he was believed to have swallowed something and his arrival at our hospital, and his level of consciousness had gradually deteriorated. On arrival, he was awake but without any visual fixations or pursuits. Moreover, the patient was hypotonic and could not maintain a sitting posture. His vital signs were normal; however, his blood oxygen levels slightly decreased during sleep. Chest and abdominal X-ray examination revealed no abnormalities. Laboratory results revealed no abnormalities except for a slightly elevated creatine kinase level (265 U/L). Computed tomography of the head showed no abnormalities. Owing to the blue tongue and disturbed consciousness, flunitrazepam poisoning was suspected, and a subsequent rapid toxicology screening kit of SIGNIFY^TM^ ER detected benzodiazepine in the urine. The patient was hospitalized to monitor his vital signs and consciousness. Two days after hospitalization, the patient regained full consciousness, and his muscle tone was normal. All individuals who had been in contact with the patient denied having benzodiazepines, and how he ingested the medicine was never ascertained, despite intensive investigation by the police.

## 3. Discussion

Poisoning is one of the leading causes of death from unintentional injury, and more than half of cases occur among children aged < 5 years who have difficulty explaining their problems verbally [1]. Drugs are also occasionally used in child abuse. Particularly, tranquilizers, including benzodiazepines, are commonly abused by parents who experience difficulty raising their children to help put their children to sleep [2]. It is difficult to recognize child drug abuse in the absence of maltreatment findings. Therefore, pediatricians should conduct case-appropriate toxicology tests to avoid missing accidental or deliberate poisoning [3]. Our patient was initially suspected of being intentionally poisoned, as the possibility of accidental ingestion was deemed unlikely given that all caregivers denied having benzodiazepines and no packages of drugs were found in his vicinity. Hence, it would have been difficult to suspect benzodiazepine poisoning without the blue tongue. As benzodiazepines had been used in drug-facilitated sexual assault, Roche reformulated its original brand, flunitrazepam, in 1997 to release a blue dye upon dissolution. Although generic versions may lack the blue dye, all flunitrazepam and triazolam tablets manufactured in Japan currently contain it regardless of the original brand or generic. In addition, several cases of accidental ingestion or overdose of flunitrazepam that were recognized based on blue-colored gastric fluid and tongue have been reported [4]. Our case highlights the utility of dyes in medicine to recognize pediatric poisoning cases suspected to involve child abuse. Oral consent was obtained for the publication of this report, and the Ethics Committee of Tokushima Red Cross Hospital approved the publication of this report (protocol number: 496).

This study had some limitations. First, rapid urine toxicology tests have limited sensitivity and specificity, which can lead to false positive results [5]. Although gas chromatography–mass spectrometry is the gold standard for toxicological analyses [5], it is not universally available in Japan. Furthermore, we could not conduct this analysis, as the remaining serum and urine samples were offered to the police. However, the positive predictive value of SIGNIFY^TM^ ER has been reported to reach as high as 96.9% (sensitivity: 67.4%; specificity 96.0%) [6]. Second, the truth about what happened to the patient remains unclear. Therefore, the patient may have ingested something other than flunitrazepam. However, the clinical symptoms and course were consistent with those of patients with benzodiazepine overdose, and we believe it was reasonable to diagnose this patient with benzodiazepine poisoning.

In conclusion, a blue tongue with disturbed consciousness can indicate benzodiazepine poisoning. Adding dyes to medicines commonly used for poisoning in children may be helpful in recognizing and preventing child abuse.

## Data Availability

The data that support the findings of this study are available from the corresponding author upon reasonable request.
